# Pneumonectomy for Primary Lung Tumors and Pulmonary Metastases: A Comprehensive Study of Postoperative Morbidity, Early Mortality, and Preoperative Clinical Prognostic Factors

**DOI:** 10.3390/curroncol30110685

**Published:** 2023-10-25

**Authors:** Konstantinos Grapatsas, Hruy Menghesha, Fabian Dörr, Natalie Baldes, Martin Schuler, Martin Stuschke, Kaid Darwiche, Christian Taube, Servet Bölükbas

**Affiliations:** 1 Department of Thoracic Surgery, West German Cancer Center, Medical Faculty, University Hospital Essen, Ruhrlandklinik, Tueschner Weg 40, 45239 Essen, Germany; hruy.menghesha@rlk.uk-essen.de (H.M.); fabian.doerr@rlk.uk-essen.de (F.D.); natalie.baldes@rlk.uk-essen.de (N.B.); servet.boeluekbas@rlk.uk-essen.de (S.B.); 2 Department of Medical Oncology, West German Cancer Center, Medical Faculty, University Hospital Essen, Hufelandstr. 55, 45147 Essen, Germany; martin.schuler@uk-essen.de; 3 Department of Radiation Oncology, West German Cancer Center, Medical Faculty, University Hospital Essen, Hufelandstr. 55, 45147 Essen, Germany; martin.stuschke@uk-essen.de; 4 Department of Pneumology, Medical Faculty, University Hospital Essen, Hufelandstr. 55, 45147 Essen, Germany; kaid.darwiche@rlk.uk-essen.de (K.D.); christian.taube@rlk.uk-essen.de (C.T.)

**Keywords:** pneumonectomy, non-small cell lung cancer, pulmonary metastases, pulmonary metastasectomy, complications, mortality, bronchus stump insufficiency, thoracic malignancy, American Society of Anesthesiologists (ASA) score

## Abstract

Background: Pneumonectomy is a major surgical resection that still remains a high-risk operation. The current study aims to investigate perioperative risk factors for postoperative morbidity and early mortality after pneumonectomy for thoracic malignancies. Methods: We retrospectively analyzed all patients who underwent pneumonectomy for thoracic malignancies at our institution between 2014 and 2022. Complications were assessed up to 30 days after the operation. Mortality for any reason was recorded after 30 days and 90 days. Results: A total of 145 out of 169 patients undergoing pneumonectomy were included in this study. The postoperative 30-day complication rate was 41.4%. The 30-day-mortality was 8.3%, and 90-day-mortality 17.2%. The presence of cardiovascular comorbidities was a risk factor for major cardiopulmonary complications (54.2% vs. 13.2%, *p* < 0.01). Postoperative bronchus stump insufficiency (OR: 11.883, 95% CI: 1.288–109.591, *p* = 0.029) and American Society of Anesthesiologists (ASA) score 4 (OR: 3.023, 95% CI: 1.028–8.892, *p* = 0.044) were independent factors for early mortality. Conclusion: Pneumonectomy for thoracic malignancies remains a high-risk major lung resection with significant postoperative morbidity and mortality. Attention should be paid to the preoperative selection of patients.

## 1. Introduction

Surgery has a major place in the treatment of thoracic malignancies. With the evolution of surgical techniques and the performance of sleeve resections, the need to perform a pneumonectomy has been reduced. However, in some cases, pneumonectomy remains the only surgical way to treat thoracic malignancies [1]. Nonetheless, pneumonectomy still remains a high-risk operation. In most cases, pneumonectomy is performed for non-small-cell lung cancer (NSCLC) [1,2,3,4]. On the other hand, the complete removal of the lung could also be needed for infectiological reasons or other malignancies. Pneumonectomy for the resection of pulmonary metastases is a rare indication [5,6,7,8,9]; for patients undergoing pneumonectomy for NSCLC, a variety of preoperative predictive factors such as age, cardiac disease, and lung function have been identified [1,2,3,4,5,10]. 

The current study aims to investigate perioperative risk factors for postoperative morbidity and early mortality for patients undergoing pneumonectomy, not only for NSCLC but also for other thoracic malignancies.

## 2. Materials and Methods

### 2.1. Study Form-Data Collection-Ethic Committee Approval

In this retrospective single-center cohort study, we investigated patients undergoing pneumonectomy for thoracic malignancies. The current study’s data were gathered from a prospective database using a retrospective analysis. With the introduction of patient data into the database, patient data were anonymized so that the patients could not be recognized anymore. 

This study was conducted according to the revised Declaration of Helsinki. Before the beginning of this study, it was approved by the local university ethics committee (Medical Faculty, University Duisburg-Essen, study-Nr.: 23-11306-BO). Due to the retrospective nature of the study and the anonymization of the patients’ data, an additional patient’s Informed Consent Statement was waived.

### 2.2. Exclusion and Inclusion Criteria

The current study included all patients who underwent pneumonectomy for NSCLC, small-cell carcinoma (SCLC), pulmonary metastases, thymic tumors, and other thoracic malignancies.

From the study, patients were excluded if they were:

- <18 years old
- patients undergoing a lung resection different than pneumonectomy
- undergoing pneumonectomy for benign or infectious disease without malignancy
- undergoing pneumonectomy because of a previous surgical complication
- undergoing extrapleural pneumonectomy for pleural mesothelioma
- undergoing pneumonectomy in the context of a lung transplant or organ retrieval for donation

### 2.3. Formation of the Study Population

In the period from February 2014 to November 2022, we identified 169 patients who underwent pneumonectomy out of 2521 patients undergoing an anatomical lung resection.

24 patients with the following diseases were excluded from the study:

- destroyed lung from pneumonia: *n* = 7
- pulmonary tuberculosis: *n* = 2
- post-stenotic pneumonia after sleeve resection: *n* = 1
- destroyed lung from aspergillosis: *n* = 1
- hemoptysis by aplasia of the pulmonary artery: *n* = 1
- anastomosis insufficiency after lung transplantation: *n* = 1
- anastomosis insufficiency after sleeve resection: *n* = 2
- pleural mesothelioma: *n* = 2
- other reasons: *n* = 7
- 145 patients were included in the study.

### 2.4. Definition of Postoperative Complications and Mortality

Morbidity or complication was defined as the appearance of a new disease unexpectedly up to 30 days or during the same hospital stay after the operation. Complications were divided into minor and major complications according to the Clavien–Dindo classification system. Clavien–Dindo grade I–II complications were defined as minor complications and required only minor or pharmaceutical treatment. Clavien–Dindo grade III–IV complications were characterized as major complications. In addition, cardiopulmonary complications with grade ≥ III in the Clavien–Dindo classification system were defined as major [11]. 

The death of any patient for any reason was characterized as mortality. Concerning mortality, patients were followed for up to 90 days after the operation. Mortality was divided into 30-day and 90-day mortality. 

### 2.5. Preoperative Staging Methods-Surgical Approach-Preoperative/Neoadjuvant Therapy

In all patients, before performing the pneumonectomy, preoperative staging was performed with positron emission tomography–computed tomography (PET-CT). In cases of suspected or positive mediastinal lymph nodes in the PET-CT, an invasive staging with bronchoscopy with endobronchial ultrasound-guided transbronchial needle aspiration (EBUS-TBNA) followed.

All patients were operated on under general anesthesia and with an open thoracotomy. In order to evaluate how surgical experience influenced the operation’s outcome, we divided the surgeon’s experience into ‘’senior consultant’’ and ‘’junior consultant/trainee’’ according to the surgeon’s previous surgical activity and the surgeon’s status in our department at the time of the operation.

Neoadjuvant chemotherapy or radiotherapy was characterized as preoperative therapy.

### 2.6. Definition of Preoperative Comorbidities

The following preoperative comorbidities were investigated: hypertension, obesity, cardiovascular comorbidity, chronic kidney disease, gastric ulcer, chronic obstructive pulmonary disease (COPD), insulin-dependent diabetes, liver disease, and myasthenia gravis.

Body Mass Index (BMI) > 30 was defined as obesity. Patients with other comorbidities that required only regular medication were characterized as other comorbidities. Patients aged >70 years were defined as elderly. COPD was classified according to lung function [12]. No patient with COPD IV was identified.

Patients regarding their smoking status were divided as current smoker, never smoker, smoker 1 month before the operation, and unknown smoking status.

### 2.7. Statistical Analysis

To evaluate connections between different parameters, Fischer’s exact test and the Mann–Whitney U test were used when appropriate. Univariate and multivariate logistic regression models were used to select independent predictors for morbidity and mortality. A *p*-value < 0.05 was considered statistically significant. A *p*-value between 0.05 and 0.07 was considered a trend. All analyses were performed using the SPSS 22.0 software (SPSS Inc., Chicago, IL, USA).

## 3. Results

### 3.1. Clinical and Surgical Characteristics of the Study Population

Most of the study population were male patients (*n* = 92, 63.4%) with a mean age of 60.6 years (range: 23–86 years). Elderly patients comprised 20% of the study population (*n* = 29). In 11.7% of the patients, a previous lung operation had been performed (*n* = 17). 

In 5 cases, a pneumonectomy was performed in patients with FEV1 29–35.6%. In all these cases, the pneumonectomy was performed on a not any more functional lung. In 1 case, an infected NSCLC that infiltrated the whole non-functioning lung with an accompanying pleural empyema was surgically treated. In the other 4 cases, a central sarcoma metastasis that produced a complete atelectasis of the lung was removed. Data concerning clinical and surgical characteristics of the study population are summarized in Table 1.

### 3.2. Oncological Characteristics of Patients Undergoing Pneumonectomy

A pneumonectomy was performed in most cases for NSCLC (84.1%). For Stages IIIA, an operation was performed for the clinical TNM-Stadium T1–2 N2, T3 N1, and T4 N0. For Stage IIIB, pneumonectomy was performed for T3 N2. For stages IIIC (*n* = 1, 0.7%), IVA (*n* = 15, 10.3%), and IVB (*n* = 3, 2.1%), a pneumonectomy in palliative intention was performed. The most frequent histological type was squamous cell carcinoma (39.3%). In 1 case, a pneumonectomy was performed because of an NSCLC recurrence.

In 5 cases with NSCLC after the pneumonectomy, a postoperative stage IB was revealed. In 1 patient, the bronchus intermedius was infiltrated, and for the complete resection of the tumor, a pneumonectomy was needed. In 1 patient, an upper bilobectomy was planned for NSCLC; a pneumonectomy was performed because of an additional destroyed lower lobe from chronic pneumonia. In another patient (*n* = 1), pneumonectomy was performed because of a lung abscess after neo-adjuvant therapy for NSCLC. In 2 patients (*n* = 2), despite neo-adjuvant chemotherapy, a pneumonectomy was needed.

The next largest group was patients with lung metastases (8.3%). Here, the most frequent histological type were patients with metastases from sarcoma. Table 2 demonstrates the oncological characteristics of this patient population. 

### 3.3. Postoperative Complications and Early Mortality after Pneumonectomy

In 41.4% (*n* = 60 patients) of the study population, at least one complication per patient was diagnosed. In almost half of these patients, a major cardiopulmonary complication was diagnosed (*n* = 29, 20%). Postoperative atrial arrhythmia was the most frequent complication. 

The 30-day mortality was rated up to 8.3%, while the 90-day mortality was 17.2% (*n* = 25). The status of 29 patients (20.0%) was unknown 90 days after the operation. Postoperative complications and mortality are described in Table 3.

### 3.4. Risk Factors for Postoperative Complications

Statistical significance was shown for elderly patients (28% vs. 13%, *p* = 0.035) and patients with cardiovascular comorbidities (25.0% vs. 10.6%, *p* = 0.021). In addition, preoperative cardiovascular comorbidities were a risk factor for postoperative major cardiopulmonary complications. A total of 54.2% (*n* = 13) of patients with cardiopulmonary comorbidities developed major cardiopulmonary complications postoperatively (vs. 13.2%, *p* < 0.01). 

Patients with ASA 4 (*p* = 0.71), smokers up to one month before the operation (*p* = 0.363), pneumonectomies in palliative intention, and extended pneumonectomy (*p* = 0.272, especially pleuropneumonectomy and sleeve pneumonectomy) and pneumonectomies performed from senior consultants showed increased postoperative morbidity. However, in these cases, no statistical significance was detected. Perioperative risk factors are summarized in Table 4. 

### 3.5. Independent Risk Factor for Postoperative Morbidity

In the logistic regression analysis, the existence of preoperative cardiovascular comorbidities showed a trend toward being an adverse independent factor for postoperative morbidity after pneumonectomy (*p*: 0.07). The logistic regression analysis is demonstrated in Table 5.

### 3.6. Risk Factors for 30-Day-Mortality

Myasthenia gravis (*p* = 0.001), deep vein thrombosis (*p* = 0.001), patients with ASA score 4 (*p* = 0.008), and bronchus stump insufficiency (*p* < 0.001) showed a statistical significance for increased 30-day-mortality after pneumonectomy. In addition, right pneumonectomy (*p* = 0.07) showed a trend for increased mortality. Patients with COPD (*p* = 0.740), obesity (*p* = 0.649), insulin-dependent diabetes (*p* = 0.559), and re-operation for bleeding (*p* = 0.166) showed increased mortality rates. However, no statistical significance was shown. These risk factors are summarized in Table 6.

### 3.7. Independent Risk Factors for Postoperative Mortality after Pneumonectomy for Thoracic Malignancies

Patients with ASA Score 4 (*p* = 0.044) and postoperative bronchus stump insufficiency (*p* = 0.029) were identified as adverse independent factors for mortality. The other two preoperative clinical factors, Myasthenia gravis, and deep vein thrombosis, were not identified as independent prognostic factors. The logistic regression analysis of risk factors for postoperative mortality after pneumonectomy for thoracic malignancies is demonstrated in Table 7.

### 3.8. Risk Factors for Bronchus Stump Insufficiency after Pneumonectomy

We identified five patients with bronchus stump insufficiency. Except for two cases, in all other three cases, the bronchus stump was covered with vital flap tissue. In two cases, the pericardial fat flap that was used for covering the bronchus stump was necrotic by the revision operation. In one case, bronchus stump insufficiency resulted from prolonged mechanical ventilation and resulted in postpneumonectomy pleural empyema.

We further investigated risk factors for bronchus stump insufficiency in the study population. An extended pneumonectomy (*p*: 0.069) showed a trend for bronchus stump insufficiency. Preoperative therapy showed an increased rate of bronchus stump insufficiency. Three patients with bronchus stump insufficiency received preoperative chemotherapy and radiation. However, no statistical significance was shown (*p*: 0.360). The risk factors for bronchus stump insufficiency are demonstrated in Table 8.

## 4. Discussion

Pneumonectomy is a disease itself. It remains a risky operation with substantial morbidity and mortality. Our study’s main findings include that bronchus stump insufficiency and the fitness of patients before the operation (ASA 4) are independent factors for early mortality. In addition, preoperative cardiovascular comorbidities were identified as a risk factor for major postoperative cardiopulmonary complications. 

Pneumonectomy is a major surgical procedure associated with a high risk of complications. Postoperative morbidity is reported between 10% and 45%, and mortality is 4–8% [1,2,3,4,5,13,14,15,16,17,18]. In our study, the 30-day mortality was up to 8.3%, and the 90-day mortality rate was 17.2%. Postoperative morbidity with at least one complication per patient was 41.4%. Major cardiopulmonary complications comprised half of these (20%). However, these rates can widely vary and depend on individual patient factors or the population being examined. A variety of perioperative factors, such as age, smoking status, COPD, poor pulmonary function, and obesity, have been identified as predictors for postoperative morbidity [13,14,15,16,17,18]. In our study, being aged > 70 years, smoking one month before the operation, palliative resection for Stage IV, and extended pneumonectomy showed an increased rate of postoperative complications. Age > 70 years has already been identified in a series of retrospective cohort studies as a factor for postoperative mortality [7]. In our study, age > 70 years was identified as an adverse prognostic factor for postoperative morbidity in the univariate analysis. However, it was not identified as an independent factor for postoperative morbidity and mortality. We believe that the careful preoperative selection of the elderly led to these results and not higher mortality. As a result, we believe that elderly patients with thoracic malignancies in good general condition should not be excluded from surgery only because of their age, but they should be preoperatively evaluated using an interdisciplinary team. In addition, concerning palliative resections, or extended pneumonectomy, it is believed that the increased postoperative morbidity was shown because of the complexity of the surgical interventions. Also, here, because of the small number of patients in the subgroups, no statistical significance was detected. Moreover, patients who smoked one month before the operation showed increased postoperative morbidity. Takenaka et al. showed similar results in their study [19]. Short-term smoking cessation did not sufficiently reduce the possibility for postoperative pulmonary complications as much as in former or never smokers. It is suggested that the best period for a patient to quit smoking would be 8 to 10 weeks before the surgery [19,20].

In 8.3% of cases, a pneumonectomy was performed for the resection of pulmonary metastases. Pneumonectomy for the resection of lung metastases is a rare indication. The surgical strategy in treating lung metastases is based on lung-sparing wedge resections and segmentectomies [6]. Hassan et al., in a large series of 760 patients undergoing pulmonary metastasectomy, identified only five patients treated with pneumonectomy [7]. The resection of pulmonary metastases in selected cases can prolong survival in selected patients [6,7,8,9,21,22,23]. The performance of pneumonectomy for lung metastases should be well-considered and well-reasoned [21,22,23]. However, the decision to perform pneumonectomy for NSCLC or lung metastases did not influence postoperative morbidity. In addition, the extension of a lung resection from lobectomy to pneumonectomy because of oncological or technical reasons did not influence the appearance of complications. Here, we believe that a selection bias could be possible, and these patients were already preoperatively adequate for a pneumonectomy. Furthermore, it was shown, also with no statistical significance, that pneumonectomies performed by senior consultants showed higher morbidity. Here, a selection bias is possible and likely, as it is believed that more complex cases were operated by senior consultants.

The appearance of a postoperative complication can be associated with preoperative comorbidities. The incidence of cardiovascular comorbidities in patients with thoracic malignancies increases with age [6,7,8,9]. The combination of lung cancer and cardiovascular disease is common in cigarette smokers. However, the influence of cardiovascular disease on the surgical treatment of thoracic malignancies is controversial [6,9,10]. Amborgi et al. showed that cardiovascular comorbidity is a negative predictor of mortality for resection of lung cancer in stages I and II [10]. Concerning pneumonectomy, Licker et al., after investigating a series of 193 patients undergoing pneumonectomy, identified preoperative cardiac morbidity as a negative predictor for early mortality [15]. However, in our case, preoperative cardiovascular comorbidities were not identified as a negative predictive factor for mortality. We believe that this relies on careful patient selection and the progress of intensive care medicine in the years that followed the study of Licker et al. An additional explanation could be that the cardiac disease was already treated, and the patients were under regular follow-up from their general practitioner or cardiologist. Moreover, preoperative cardiovascular comorbidity was a negative prognostic factor for major postoperative cardiopulmonary complications and showed a trend for complications generally. These postoperative complications are a result of hemodynamic changes after pneumonectomy. Cardiac output and stroke volume are lower after pneumonectomy than before, but heart rate response could be unaltered. This pattern of responses suggests that increases in left and right ventricular afterload may contribute to a reduction in cardiac output. We believe these hemodynamic changes in an already disadvantaged cardiac system led to these major complications in our study [6,24]. 

As mentioned above, a patient’s preoperative general status and pre-existing comorbidities are crucial criteria in selecting patients for surgery. This selection is more important in our case if the complete resection of the lung is planned. Patients with preoperative clinical risk factors such as Myasthenia gravis and deep vein thrombosis, which as conditions display the suspicion of reduced general condition, should be carefully evaluated for surgery. Different scores evaluate the patient’s preoperative condition and some also the possibility of postoperative complications [25,26,27]. The ASA physical status classification system helps to assess the fitness of patients before the operation [28]. Maret et al. have already identified ASA > 2 as an independent factor for major cardiopulmonary complications and mortality after pneumonectomy [4]. In addition, Mazella et al. identified ASA score as an independent factor for ARDS after pneumonectomy [29]. Similarly, in our study, the ASA score, particularly for patients with an ASA score of 4, was an independent score for mortality.

Postoperative bronchus stump insufficiency was identified as an independent risk factor for mortality after pneumonectomy. The rates for bronchus stump insufficiency after pneumonectomy range from 0.5 to 4.4%. Increased rates for bronchus stump insufficiency are found after a right pneumonectomy and range from 9% to 14%. Mortality after bronchus stump insufficiency ranges between 25% and 71%. As preoperative risk factors for bronchus stump insufficiency have been identified, preoperative radiotherapy, diabetes, right pneumonectomy, postoperative mechanical ventilation, tumor residuals on the bronchus stump (R1/R2-resection), and radical lymphadenectomy [16,17,18]. In our study, patients with preoperative chemotherapy, radiation, and extended pneumonectomy showed increased rates of postoperative bronchus stump insufficiency after pneumonectomy. However, in these cases, no statistical significance was shown. We believe this lies in the small number of patients in each group. However, in 80% of these cases (4 out of 5 patients), the bronchus stump after the pneumonectomy was not covered adequately. We believe that covering the bronchus stump with vital tissue might be important for the successful healing of the bronchus stump. For this task, several vital flaps have been described. We recommend covering bronchus stumps with vital flaps (e.g., pericardial, intercostal muscle), especially in cases of a right pneumonectomy. Bronchus stump insufficiency after a pericardial fat flap coverage is reported to be up to 4.9%. However, the manner of the covering could be modified depending on the experience and preference of the surgeon [30,31,32]. Taghavi showed no bronchus stump insufficiency after pneumonectomy and covering of the bronchus stump with a pedicled pericardial flap [33].

Preoperative (adjuvant) therapy can provide better overall survival and disease-free interval with a higher pathological complete response rate or major pathologic response by shrinking tumors [34]. In this way, the performance of pneumonectomy could be avoided. Misumi et al. showed that in a selected case for centrally located NSCLC, the performance of pneumonectomy after neoadjuvant therapy could be avoided. However, this study included highly selected patients without invasion to the carina or right-or-left main trunk of the pulmonary artery or vein at pretreatment [35]. As mentioned above, preoperative therapy is, in some studies, an identified factor for bronchus stump insufficiency, postoperative mortality, and morbidity [16,17,18]. Weder et al., in a multicenter study, suggested that patients after neoadjuvant therapy should not be excluded from a curative pneumonectomy. In the same study, a 38% 5-year survival was achieved [36].

### Limitations

The current study is limited by its retrospective nature. Data in this study were retrospectively extracted from an anonymized institutional database. Missing information could theoretically influence the results of this study. In addition, a selection bias is possible. Possibly, patients with untreated preoperative comorbidities or elderly patients in reduced general condition were excluded from the pneumonectomy procedure. Furthermore, an additional limitation is the variety of thoracic primary tumors that were included. What is more, the limited number of patients in some patient groups could have influenced the statistical result. Moreover, the classification of COPD was made, taking into account only the lung function. Furthermore, because of the limited number of patients in the current study and in order to avoid repeating results, risk factors for mortality were calculated only for 30-day mortality and not also for 90-day mortality. However, our study contains real-life clinical data and depicts the everyday clinical praxis in a large Thoracic Surgery Department. For this reason, we believe that patients who need a major lung resection such as pneumonectomy should not be excluded from a potentially curative resection only because of their preoperative comorbidities, but they should be carefully evaluated.

## 5. Conclusions

Pneumonectomy for thoracic malignancies is still a major surgery with increased postoperative morbidity and mortality. The patient’s condition before surgery is crucial for selecting patients for pneumonectomy. Preoperative clinical conditions such as cardiovascular disease, Myasthenia gravis, and deep vein thrombosis that influence the patient’s general status should be carefully evaluated in patients’ selection.

## Figures and Tables

**Table 1:** Clinical and surgical characteristics of the study population.

Clinical Factors	*n* Patients (%)
Gender	
male	92 (63.4%)
female	53 (36.6%)
Age (years)	
Mean	60.6276
Median	62.00
Range	23.00–86.00
Elderly patients (age > 70 years)	29 (20%)
Preoperative comorbidities	
At least one comorbidity per patient	115 (79.9%)
Hypertension	62 (42.8%)
Obesity (BMI > 30)	18 (12.4%)
Cardiovascular comorbidity	24 (16.6%)
Chronic kidney disease	2 (1.4%)
Gastric ulcer	3 (2.1%)
COPD I–III	43 (29.7%)
-COPD I	7 (16.3%)
-COPD II	32 (74.4%)
-COPD III	4 (9.3%)
Insulin-dependent diabetes	7 (4.8%)
Liver disease	5 (3.4%)
Myasthenia gravis	1 (0.7%)
Other comorbidities	79 (54.5%)
Smoking history	
-current smoker	38 (26.2%)
-never smoker	14 (9.7%)
-smoker 1 month before the operation	67 (46.2%)
-unknown	26 (17.9%)
Preoperative lung function	
FEV1%	
Mean	72.86
Median	72.00
Range	29.00–147.00
DLCO%	
Mean	60.25
Median	59.10
Range:	33.40–100.70
Surgical characteristics	
Previous lung operations	17 (11.7%)
Pneumonectomy with additional resection:	
-Only pneumonectomy	56 (38.6%)
-with atrial resection	10 (6.9%)
-completion-pneumonectomy	2 (1.4%)
-with diaphragm resection	3 (2.1%)
-Intrapericardial	51 (35.2%)
-Pleuropneumonectomy	11 (7.6%)
-Sleeve pneumonectomy	10 (6.9%)
-with SVC resection and reconstruction	2 (1.4%)
Side of pneumonectomy:	
-left	73 (50.3%)
-right	72 (49.7%)
Preoperative plan for lobar resection with intraoperative extension to pneumonectomy	23 (15.9%)
Lymphadenectomy:	
Not performed	20 (13.8%)
performed	125 (86.2%)

BMI: Body-Mass-Index, COPD: Chronic Obstructive Pulmonary Disease, FEV1: Forced Expiratory Volume in 1 s, DLCO: Diffusing capacity for carbon monoxide, SVC: superior vena cava.

**Table 2:** Oncological characteristics of patients undergoing pneumonectomy.

Variable/ Oncological Characteristics	*n* Patients, %
Preoperative treatment:	
-none	64 (44.1%)
-chemotherapy	41 (28.3%)
-chemotherapy + radiation	38 (26.2%)
-unknown	2 (1.4%)
Type of thoracic malignancies:	
-NSCLC	122 (84.1%)
-SCLC	1 (0.7%)
-pulmonary metastases	12 (8.3%)
-thymic tumors	1 (0.7%)
-other thoracic malignancies	9 (6.2%)
Pulmonary metastases:	
-CRC	3
-soft-tissue-/osteosarcoma	7
-germ cell tumor	1
-breast cancer	1
NSCLC	
Preoperative T-stage (cT)	
cTx	1 (0.7%)
cT1	8 (5.5%)
cT2	20 (13.8%)
cT3	31 (21.4%)
cT4	56 (38.6%)
unknown	29 (20%)
Preoperative lymph node status (cN)	
cN0	51 (35.2%)
cN1	22 (15.2%)
cN2	36 (24.8%)
cN3	6 (4.1%)
cNx	1 (0.7%)
unknown	29 (20%)
M-status	
cM0	99 (68.3%)
cM1	17 (11.7)
unknown	29 (20%)
Postoperative T-stage (pT)	
pTx	10 (6.9%)
pT1	11 (7.6%)
pT2	33 (22.8%)
pT3	34 (23.4%)
pT4	27 (18.6%)
unknown	30 (20.7%)
Postoperative lymph node status (pN)	
pN0	38 (26.2%)
pN1	55 (37.9%)
pN2	21 (14.5%)
pN3	1 (0.7%)
unknown	30 (20.7%)
Tumor stadium UICC 8	
Recurrence of NSCLC	1 (0.7%)
IB	5 (3.4%)
IIA	2 (1.4%
IIB	33 (22.8%)
IIIA	40 (27.6%)
IIIB	15 (10.3%)
IIIC	1 (0.7%)
IVA	15 (10.3%)
IVB	3 (2.1%)
unknown	30 (20.7%)
Histology of NSCLC:	
-adenocarcinoma	45 (31.0%)
-large cell	4 (2.8%)
-mixed	7 (4.8%)
-neuroendocrine	7 (4.8%)
-squamous cell	57 (39.3%)
-unknown	25 (17.2%)

NSCLC: Non-small-cell lung cancer, SCLC: Small-cell carcinoma, CRC: Colorectal cancer, UICC: Union for International Cancer Control.

**Table 3:** Postoperative complications and early mortality after pneumonectomy.

Variable/ Postoperative Complication	*n* Patients, %
At least one complication per patient	60 (41.4%)
Major complications	54 (37.2%)
Minor complications	6 (4.1%)
major cardiopulmonary complication per patient	29 (20.0%)
-atrial arrhythmia postoperative	11 (7.6%)
-cardiac failure	3 (2.1%)
-initial ventilation > 48 h	9 (6.2%)
-pneumonia	3 (2.1%)
-ventricular arrhythmia	6 (4.1%)
-bronchus stump insufficiency	5 (3.4%)
-pleural empyema	2 (1.4%)
re-operation for bleeding	10 (6.9%)
chylothorax	1 (0.7%)
delirium	1 (0.7%)
deep vein thrombosis	1 (0.7%)
dysphagia/aspiration	1 (0.7%)
dysphonia	1 (0.7%)
chest wall hematoma	2 (1.4%)
multisystem failure	4 (2.8%)
recurrent nerve pulse	9 (6.2%)
renal failure	4 (2.8%)
unexpected admission to the ICU	4 (2.8%)
wound infection	2 (1.4%)
Mortality	
30-day mortality	12 (8.3%)
90-day-mortality	25 (17.2%)

ICU: intensive care unit.

**Table 4:** Univariate analysis of patient-specific and procedural risk factors for postoperative complications.

Patient-Specific Risk Factor	No Postoperative Complication (*n* Patients, %)	Postoperative Complications (*n* Patients, %)	*p*-Value
Preoperative comorbidity	63 (75%)	52 (86.7%)	0.085
Cardiovascular comorbidity	9 (10.6%)	15 (25.0%)	0.021
Elderly (age > 70 years)	12 (14.1%)	17 (28.3%)	0.035
Obesity	13 (15.3%)	5 (8.3%)	0.211
ASA score			
ASA 3	28 (70.0%)	20 (64.5%)	0.710
ASA 4	3 (7.5%)	5 (16.1%)	
ECOG			
ECOG 2	4 (6.6%)	3 (8.1%)	0.624
ECOG 3	1 (1.6%)	0 (0%)	
Hypertension	37 (43.5%)	25 (41.7%)	0.823
Chronic kidney disease	2 (2.4%)	0 (0%)	0.232
Gastric ulcer	1 (1.2%)	2 (3.3%)	0.369
COPD I–III	27 (31.8%)	16 (26.7%)	0.233
-COPD I	3 (11.1%)	4 (25.0%)	0.233
-COPD II	23 (85.2%)	9 (56.2%)	0.036
-COPD III	1 (3.7%)	3 (18.8%)	0.101
Insulin-dependent diabetes	4 (4.7%)	3 (5.0%)	0.935
Liver disease	2 (2.4%)	3 (5.0%)	0.390
Myasthenia Gravis	0 (0%)	1 (1.7%)	0.232
Other comorbidity	43 (50.6%)	36 (60.0%)	0.262
Urgent operation	5 (5.9%)	3 (5.0%)	0.946
Previous lung surgery	11 (12.9%)	6 (10.2%)	0.612
Systematic lymph node dissection	19 (22.4%)	13 (21.7%)	0.455
Preoperative treatment			
-chemotherapy	24 (28.2%)	17 (28.8%)	0.391
-chemotherapy and radiotherapy	26 (30.6%)	12 (20.3%)	
Smoker one month before the operation	34 (40.0%)	33 (55.0%)	0.363
Type of tumor			
-NSCLC	75 (88.2%)	47 (78.3%)	0.230
-SCLC	0 (0.0%)	1 (1.7%)	
-Pulmonary metastases	7 (8.2%)	5 (8.3%)	
-Thymic malignancies	0 (0.0%)	1 (1.7%)	
-Other thoracic malignancies	3 (3.5%)	6 (10.0%)	
NSCLC			
Preoperative tumor size (cT)			
cTx	1 (1.4%)	0 (0%)	0.236
cT1	4 (5.7%)	4 (8.7%)	
cT2	8 (11.4%)	12 (26.1%)	
cT3	21 (30.0%)	10 (21.7%)	
cT4	36 (51.4%)	10 (21.7%)	
Preoperative lymph node status (cN)			
cNx	1 (1.4%)	0 (0%)	0.220
cN0	31 (44.3%)	20 (43.5%)	
cN1	14 (20.0%)	8 (17.4%)	
cN2	23 (32.9%)	13 (28.3%)	
cN3	1 (1.4%)	5 (10.9%)	
Preoperative M-status			
cM0	61 (85.9%)	38 (82.6%)	0.574
cM1	9 (12.7%)	8 (17.4%)	
Postoperative tumor size (pT)			
pTx	9 (13.0%)	1 (2.2%)	0.105
pT1	8 (13.0%)	2 (4.3%)	
pT2	19 (27.5%)	14 (30.4%)	
pT3	17 (24.6%)	14 (37.0%)	
pT4	15 (21.7%)	12 (26.1%)	
Postoperative lymph node status (pN)			
pN0	26 (37.7%)	12 (26.1%)	0.343
pN1	30 (43.5%)	25 (54.3%)	
pN2	13 (18.8%)	8 (17.4%)	
pN3	0 (0.0%)	1 (2.2%)	
Stadium I-IV			
Recurrence of NSCLC	1 (1.4%)	0 (0.0%)	0.952
IB	3 (4.3%)	2 (4.3%)	
IIA	1 (1.4%)	1 (2.2%)	
IIB	20 (29.0%)	13 (28.3%)	
IIIA	25 (36.2%)	15 (32.6%)	
IIIB	9 (13.0%)	6 (13.0%)	
ΙΙΙC	0 (0.0%)	1 (2.2%)	
IVA	8 (11.6%)	7 (15.2%)	
IVB	2 (2.9%)	1 (2.2%)	
Histological tumor group			
-Adenocarcinoma	30 (41.1%)	15 (31.9%)	0.573
-Large cell carcinoma	3 (4.1%)	1 (2.1%)	
-Mixed	3 (4.1%)	4 (8.5%)	
-Neuroendocrine	3 (4.1%)	4 (8.5%)	
-Squamous cell	34 (46.6%)	23 (48.9%)	
Extended pneumonectomy	49 (57.6%)	40 (66.7%)	0.272
Type of pneumonectomy			
-Only pneumonectomy	36 (42.4%)	20 (33.3%)	0.149
-With atrial resection	8 (9.4%)	2 (3.3%)	
-Completion-pneumonectomy	1 (1.2%)	1 (1.7%)	
-Diaphragm resection	1 (1.2%)	2 (3.3%)	
-Intrapericardial	30 (35.3%)	21 (35%)	
Pleuropneumonectomy	3 (3.5%)	8 (13.3%)	
-Sleeve resection	4 (4.7%)	6 (10.0%)	
-SVC resection with reconstruction	2 (2.4%)	0 (0.0%)	
Operation side			
-Left	44 (51.8%)	29 (48.3%)	0.684
-Right	41 (48.2%)	31 (51.7%)	
Preoperative planed lobectomy	16 (18.8%)	7 (11.7%)	0.245
Surgeon’s experience			
-senior consultant	29 (34.1%)	27 (45.0%)	0.319
-junior consultant/trainee	30 (35.3%)	15 (25.0%)	
-unknown	26 (30.6%)	18 (30.0%)	
Preoperative therapy			
Preoperative therapy			
-no	34 (40.0%)	31 (51.7%)	0.16
-yes	51 (60.0%)	29 (48.3%)	

ASA: American Society of Anesthesiologists, ECOG: Eastern Cooperative Oncology Group, FEV1: Forced Expiratory Volume in 1 s, DLCO: Diffusing capacity for carbon monoxide, COPD: Chronic Obstructive Pulmonary Disease, NSCLC: Non-small-cell lung cancer, SCLC: Small-cell carcinoma, SVC: superior vena cava.

**Table 5:** Logistic regression analysis of factors associated with postoperative complications after pneumonectomy for malignancies.

Variable	OR	95% CI	*p*-Value
Elderly (age > 70 years)	1.963	0.824–4.674	0.128
Cardiovascular comorbidities	2.331	0.909–5.976	0.07

**Table 6:** Risk factors for early (30-day) mortality.

Variable/ Comorbidity	No 30-Day-Mortality (*n* Patients, %)	30-Day-Mortality (*n* Patients, %)	*p*-Value
Preoperative clinical risk factors			
Age			
Age < 70 years	104 (90.4%)	11 (9.6%)	0.287
elderly (age > 70)	28 (96.6%)	1 (3.4%)	
Weight			
Normal weight	116 (92.1%)	10 (7.9%)	0.649
obesity (BMI > 30)	16 (88.9%)	2 (11.1%)	
Comorbidity preoperative			
No	25 (86.2%)	4 (13.8%)	0.240
Yes	106 (93.0%)	8 (7.0%)	
preoperative cardiovascular comorbidity			
no	109 (90.8%)	11 (9.2%)	0.418
yes	23 (95.8%)	1 (4.2%)	
Postoperative complication			
No	78 (91.8%)	7 (8.2%)	0.959
Yes	54 (91.5%)	5 (8.5%)	
ASA score			
-unknown	69 (94.5%)	4 (5.5%)	0.008
-ASA score 1–3	58 (92.1%)	5 (7.9%)	
-ASA score 4	5 (62.5%)	3 (37.5%)	
COPD			
No	94 (71.2%)	8 (66.7%)	0.740
Yes	38 (28.8%)	4 (33.3%)	
Insulin-dependent diabetes			
No	126 (92.0%)	11 (8.0%)	0.559
yes	6 (85.7%)	1 (14.3%)	
Myastehnia Gravis			
No	132 (92.3%)	11 (7.7%)	0.001
Yes	0 (0.0%)	1 (100.0%)	
deep vein thrombosis			
no	132 (92.3%)	11 (7.7%)	0.001
yes	0 (0.0%)	1 (100.0%)	
Surgery depends on risk factors			
extended pneumonectomy			
no	52 (92.9%)	4 (7.1%)	0.680
yes	80 (90.9%)	8 (9.1%)	
bronchus stump insufficiency			
no	130 (93.5%)	9 (6.5%)	<0.001
yes	2 (40.0%)	3 (60.0%)	
reoperation for bleeding			
no	124 (92.5%)	10 (7.5%)	0.166
yes	8 (80.0%)	2 (20.0%)	
Side of pneumonectomy			
Right	69 (95.8%)	3 (4.2%)	0.070
Left	63 (87.5%)	9 (12.5%)	
Surgeon’s experience			
-senior consultant	49 (89.1%)	6 (10.9%)	0.218
-junior consultant/trainee	40 (88.9%)	5 (11.1%)	
-unknown	43 (97.7%)	1 (2.3%)	
Oncological therapy			
Preoperative therapy			
-No	60 (45.5%)	5 (41.7%)	0.80
-yes	72 (54.5%)	7 (58.3%)	

ASA: American Society of Anesthesiologists, COPD: Chronic Obstructive Pulmonary Disease, FEV1: Forced Expiratory Volume in 1 s.

**Table 7:** Logistic regression analysis of factors associated with early postoperative mortality after pneumonectomy for thoracic malignancies.

Variable	OR	95% CI	*p*-Value
Bronchus stump insufficiency	11.883	1.288–109.591	0.029
ASA 4	3.023	1.028–8.892	0.044
Myasthenia gravis	-	-	1.000
Deep vein thrombosis	-	-	1.000

ASA: American Society of Anesthesiologists.

**Table 8:** Risk factors for bronchus stump insufficiency after pneumonectomy.

Risk Factor	No Bronchus Stump Insufficiency (*n* Patients, %)	Bronchus Stump Insufficiency (*n* Patients, %)	*p*-Value
extended pneumonectomy	83 (59.7%)	5 (100.0%)	0.069
initial ventilation > 48 h	1 (0.7%)	0 (0.0%)	0.849
preoperative chemotherapy + radiation	34 (24.6%)	3 (60.0%)	0.360
Right pneumonectomy	70 (50.4%)	2 (40.0%)	0.649

## Data Availability

The data presented in this study are available upon request from the corresponding author.
